# Advancing Politeness and Assertive Communication Through Tone of Voice in Crisis Team Situations: Pre-Post Acoustic Analysis Study of Team and Strategies to Enhance Performance and Patient Safety (TeamSTEPPS) Virtual Simulation for Interprofessional Education in Health Care Undergraduate Students

**DOI:** 10.2196/66988

**Published:** 2025-04-21

**Authors:** Sujinat Jitwiriyanont, Rattanasuwan Rawan, Khuansiri Narajeenron

**Affiliations:** 1 Department of Linguistics and Center of Excellence in Southeast Asian Linguistics Faculty of Arts Chulalongkorn University Bangkok Thailand; 2 Department of Foreign Languages Faculty of Liberal Arts Rajamangala University of Technology Krungthep Bangkok Thailand; 3 Department of Emergency Medicine Faculty of Medicine Chulalongkorn University and King Chulalongkorn Memorial Hospital Bangkok Thailand

**Keywords:** virtual simulation, TeamSTEPPS, acoustic analysis, prosodic features, interprofessional education, politeness, assertive communication, tone of voice, confident, respect

## Abstract

**Background:**

Effective interprofessional communication, including politeness, respect for coworkers, and self-control, is crucial in emergency care. These values are emphasized by both Thai and US cultures. Notably, nurses place greater significance on respect and self-control than physicians, underscoring the need for physicians to recognize and adopt these attributes, especially in interactions with nursing staff. To develop these competencies, interprofessional education (IPE) programs are essential, with simulation-based IPE, particularly virtual simulations, showing promise in enhancing teamwork and communication. However, research on the tone of voice in emergency communication is limited, especially in Thailand, where standardized IPE curricula are lacking.

**Objective:**

This study aimed to assess the effectiveness of Team and Strategies to Enhance Performance and Patient Safety (TeamSTEPPS) virtual simulation IPE, using a 3D computer-based or virtual reality (VR) approach, in enhancing interprofessional communication among health care students, focusing on politeness and assertiveness in the tone of voice.

**Methods:**

An experimental design was used with 30 health care students from 5 disciplines, including medical, nursing, medical technology, radiological technology, and pharmacy students. Participants were recorded during pretraining and posttraining TeamSTEPPS sessions. Acoustic analysis focused on 3 cues: duration, intensity, and fundamental frequency (F0). Duration measured the length of utterances, whereas intensity (loudness) and F0 (pitch) were analyzed using parameters, such as maximum, minimum, mean, SD, and range. In total, 5663 utterances were analyzed, providing a dataset for identifying significant shifts in vocal delivery after training. Using the Wilcoxon signed-rank test, these 11 acoustic parameters were extracted and statistically analyzed to compare pre- and posttraining differences.

**Results:**

Significant improvements in the tone of voice were observed. Medical and nursing students exhibited changes in pitch (SD and mean of F0; *P*<.001) and loudness (mean of intensity, *P*<.001), suggesting more attentive communication. The increased utterance duration in core team members suggests that they engaged in more elaborate information sharing and verification, critical for patient safety. Medical technology and radiological technology students showed reduced pitch (mean of F0; *P*<.05 in medical technology students and *P*<.01 in radiological students) and intensity (mean of intensity; *P*<.01 in medical technology students), reflecting calmer, more controlled communication. Pharmacy students showed minor changes.

**Conclusions:**

TeamSTEPPS virtual simulation IPE, using a 3D computer-based or VR approach, effectively enhances interprofessional nonverbal communication by improving key acoustic features related to politeness and assertiveness within the Thai culture context. Medical and nursing students showed the most notable gains, whereas quieter more controlled communication styles emerged among the other groups. The results demonstrate the ways in which vocal modulations can reflect role-specific responsibilities and interpersonal sensitivity in clinical interactions. These findings highlight the significance of tailored virtual simulation IPE programs for improving teamwork and patient outcomes across health care disciplines.

## Introduction

In emergency situations, interprofessional team communication is crucial not only for the quality and safety of patient care but also for the well-being and mental health of health care providers. Research focusing on the professionalism of emergency physicians in Thailand and the United States has highlighted essential competencies for effective teamwork, including being a collaborative team player, demonstrating effective communication, showing respect for colleagues, and maintaining politeness. These competencies have been assessed through the perceptions of faculty members, physicians, nurses, residents, and medical students [1].

In health care, mutual respect is shown to enhance communication, as highlighted by Robinson et al [2] and Weller et al [3], who link respect to better teamwork and patient safety, while Stecker [4] emphasizes the negative impact of disrespect. In linguistics, according to the Politeness Theory proposed by Brown and Levinson [5], politeness involves preserving the listener's “face”—either positive face (the desire to be liked and appreciated) or negative face (the desire for autonomy and freedom from imposition). In this paper, politeness is defined from a linguistic perspective, focusing on how language is used to convey respect and consideration.

Politeness, as a linguistic device for showing respect, is supported by Brown and Levinson's [5] theory, which explains how polite language preserves the listener's face and fosters respectful communication. In Thai society, politeness through honorifics, speech levels, and vocal tones reinforces respect, thus facilitating effective interpersonal communication, much like in professional health care settings.

In communication, especially in high-stakes environments such as the emergency department (ED), verbal content alone is frequently insufficient to completely convey meaning. Mehrabian’s Communication Theory [6] highlights the larger role of nonverbal cues, including the tone of voice and facial expressions, in communication than words. In fact, only 7% of meaning comes from the actual words, whereas 38% is derived from the tone of voice and 55% from facial expressions and body language. In the fast-paced ED, where time-sensitive decisions are made, confusion or delays in critical actions can arise owing to misalignment between verbal and nonverbal cues. To prevent misunderstandings, improve teamwork, and enhance patient safety, ensuring that tone and body language match the verbal message is essential.

Several studies have emphasized the role of prosody in shaping perceptions of politeness [7-9]. Navarro and Nebot [10] highlighted the significance of prosodic cues in conveying politeness [11-13]. In linguistic terms, prosody involves the study of pitch (fundamental frequency), loudness (intensity), and duration, all of which contribute to the tone of voice. Pitch, measured in hertz, refers to how high or low a voice sounds, with fundamental frequency (F0) as the primary measure. Loudness, measured in decibels, reflects the perceived strength of a voice, whereas duration influences the rhythm and flow of speech.

Prosody, including pitch, loudness, and duration, shapes perceptions of politeness by influencing vocal tone. These acoustic features are analyzed using parameters like mean, SD, and range. While studies on Thai politeness are limited, research on Japanese, Korean, and Chinese [14-16] provides useful insights for forming hypotheses about Thai prosodic politeness. Together, these acoustic properties significantly shape how politeness, respect, or assertiveness is perceived in communication.

Polite speech, as investigated in linguistics, is complex and varies across cultures and generations. Jitwiriyanont and Saisuwan [17] reported that Thai speakers, especially those from Generation Z, tend to use a higher pitch; however, F0 variability—not the sole overall pitch—was key to how politeness was perceived across social groups. This finding emphasizes the significance of prosodic features in fostering respectful communication, especially in ED settings, where the stakes for effective teamwork are high. However, the tone of voice is also culture-specific. What works for English-speaking professionals may not directly apply in Thai settings. In the case of interprofessional communication in Thailand, no previous studies have explored the role of prosodic features, presenting a significant research gap.

Given the categorization of physicians and nurses as the core team and the other 3 groups (pharmacy, medical technology, and radiological technology) as the support team, it is hypothesized that physicians and nurses will exhibit greater pitch variability (mean of F0) and intensity range*,* reflecting more dynamic and attentive communication styles. This is due to their leadership and decision-making roles in emergency scenarios, which require assertive yet polite communication. Conversely, the support team members are expected to demonstrate more controlled and consistent vocal patterns*,* with lower pitch variability and intensity, reflecting a more reserved communication style aligned with their assisting roles. These differences in prosodic features are anticipated to enhance team dynamics by clearly signaling leadership and supportive roles during high-stakes interactions.

In Thai and United States ED cultures, politeness, respect, and self-control are crucial to successful interprofessional collaboration. Although patients highly value polite and respectful communication, nurses, in particular, emphasize the significance of respect and self-control more than physicians do. This finding highlights the need for physicians to be more attuned to these aspects, especially when collaborating with nursing staff. Understanding and practicing respect, self-control, and politeness are crucial for fostering a collaborative work environment that ultimately benefits patient care and teamwork in high-pressure emergency settings [1].

In Thailand, to date, no standardized interprofessional education (IPE) curriculum for clinical health students to clearly and comprehensively practice effective communication, particularly in using the tone of voice for alerting and conveying essential information, is available. To address this gap, our research team has developed a virtual simulation IPE scenario for coronavirus disease 2019 pneumonia and recorded the audio for investigation. The aim is to evaluate whether virtual simulation IPE using Team and Strategies to Enhance Performance and Patient Safety (TeamSTEPPS) can effectively enhance teamwork communication skills and serve as a tool for developing the tone of voice for clinical students, including medical, nursing, pharmacy, radiological technology, and medical technology students.

TeamSTEPPS, a widely adopted framework for improving teamwork and communication in health care, emphasizes the significance of clear and respectful communication. Students in medical settings are routinely trained using this framework, making communication a key focus. To better understand the impact of TeamSTEPPS, this study aimed to compare the tone of voice used by students before and after TeamSTEPPS training. Specifically, this study explored whether their tone of voice positively changed from training to testing rounds, contributing to more effective and respectful communication.

Here, a comparative analysis of prosodic features in pre- and posttraining recordings from simulated ED scenarios was conducted. This study focused on interprofessional communication among Thai health care professionals. By comparing recordings from training and testing rounds, this study aimed to assess the effectiveness of TeamSTEPPS virtual simulation in enhancing interprofessional communication among undergraduate health care students in crisis team situations, focusing on politeness and assertiveness in the tone of voice, particularly in a Thai-speaking context.

## Methods

### Recruitment

This study recruited 5 groups of undergraduate (clinical level) students from Chulalongkorn University, comprising 6 medical students, 6 nursing students, 6 medical technology students, 5 radiological technology students, and 6 pharmacy students. All of them participated in pretraining sessions, TeamSTEPPS training, and posttraining sessions. Following the Institution Research Ethics Committee, they were given an information sheet detailing the study’s aim, the discomfort they might experience, the right to withdraw from the study with no subsequent effect, and the confidentiality of their personal information. All of the participants had given written consent before participating in the study.

### Sample Size

Due to challenges during the COVID-19 pandemic, we only recruited 29 participants into the study. The average effect size from all of our analyses yielded a power of 0.664. Although the power does not meet the conventional threshold, it serves as preliminary evidence to support the feasibility and potential significance of expanding this research in future studies with a larger sample size. The analysis included 5663 utterance samples. The number of sample utterances and number of participants in the study are presented in Table 1.

**Table 1:** Number of utterance samples produced by participants in the sessions grouped by faculty.

Students	Number of utterance samples	Proportion	Number of participants
Medical	2377	42.14	6 (3 male, 3 female)
Nursing	2028	35.95	6 (1 male, 5 female)
Pharmacy	519	9.2	6 (3 male, 3 female)
Medical technology	502	8.9	6 (3 male, 3 female)
Radiological technology	215	3.81	5 (2 male, 3 female)

### TeamSTEPPS-Based Simulation for Interprofessional Education

This study implemented a TeamSTEPPS-based training program using a 3D computer-based or virtual reality (VR) simulation–based IPE approach. The training focused on managing a 70-year-old male patient with chronic obstructive pulmonary disease (COPD), diabetes mellitus, hypertension, and dyslipidemia, who presented with acute dyspnea for 2 hours before arrival and impending respiratory failure due to COVID-19 pneumonia complicated by hospital-acquired pneumonia and hyperkalemia. His medical history included anaphylaxis to third-generation cephalosporins, a 40-pack-year smoking history, and the use of Berodual MDI. Two months previous, he had been hospitalized with community-acquired pneumonia and a COPD exacerbation, requiring intensive care unit admission for 2 weeks due to a difficult airway, before successful extubation and discharge.

The training session lasted 30 to 60 minutes and covered all 5 TeamSTEPPS core domains: team structure, communication, leadership, situation monitoring, and mutual support. A flipped classroom model was used, requiring participants to engage in self-regulated preclass learning through a massive open online course on TeamSTEPPS principles. Completion of an assessment and certification was mandatory before attending the live session. Each training session included an interprofessional team of clinical-level students, consisting of 1 medical student, 2 nursing students, 1 medical technology student, 1 radiology student, and 1 pharmacy student. The in-class training was facilitated by an interprofessional team of 2 to 5 instructors from different health care disciplines, who guided participants through the simulation and co-led the debriefing session.

### Speech Data Collection

Participants took part in one virtual simulated ED scenario, which was developed as a part of the “Interprofessional Medical Education Model Development for Enhancing Emergency Patients Safety through Online Virtual Simulation-Based Learning Platform” project. One session required 6 players (one from each medical field, except for 2 nurses) and lasted for approximately 30-60 minutes. In the given ED scenario, participants were required to communicate and help each other in treating an older adult patient with polypharmacy presenting with acute respiratory failure symptoms with critical hyperkalemia. In the session, each player assumed a role through a simulated character. Each simulated session was videotaped and audio-recorded.

**Figure 1:**
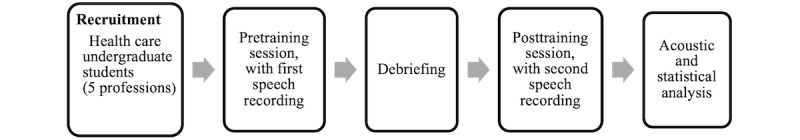
Procedures for data collection and processing in this study. The TeamSTEPPS virtual simulation-based interprofessional education training lasted approximately 30-60 minutes.

Recordings from pretraining and posttraining sessions were extracted and digitized at a 44.1-kilo hertz sampling frequency, represented by 16 bits per sample, and stored on a PC for acoustic analysis. Audio data were collected using Audacity version 3.4.2, with desktop and VR headset microphones. For desktop microphones, precise positioning, distance checks, and mic-level adjustments were performed to ensure data integrity, whereas VR headset microphones were used to minimize interference from individuals outside the professional disciplines. The TeamSTEPPS virtual simulation-based interprofessional education (SimBIE) training lasts approximately 30-60 minutes. Figure 1 describes the procedure to collect data for the study.

### Acoustic Analysis

Three acoustic cues were extracted from recordings during pretraining and posttraining sessions. Duration is the first acoustic cue. The parameter utterance duration represents the duration of the utterance the participants speak during the training. Each utterance is determined by the presence of speech signals from the onset to the offset followed by a >30-millisecond silence. The recordings marked utterance boundaries using a Praat [18] script; however, one of the researchers and a research assistant manually adjusted the boundaries after automatic marking by the script. The second acoustic cue is loudness, characterized by the intensity of the waveform in the sound wave. The parameters for intensity include maximum, minimum, average, SD, and range. Maximum and minimum were collected from the maximum and minimum points of intensity in the utterance. Average, SD, and range were then calculated for the data. The third acoustic cue is F0, characterized by the rate of vibration of the vocal folds when producing sounds. The collection and the calculation for average, SD, and range of F0 are similar to that of intensity. Collecting F0 and intensity was automatic, with the use of a Praat script. The Praat script was set to extract intensity every 10 milliseconds of the utterance duration and F0 every 10 milliseconds within a range of 20 to 600 hertz. Overall, 11 acoustic parameters were extracted, including utterance duration, minimum of intensity, maximum of intensity, mean of intensity, SD of intensity, range of intensity, minimum of F0, maximum of F0, mean of F0, SD of F0, and range of F0.

### Data Exclusion

Utterances that appeared to the researchers to be self-correction and filler expressions were excluded from the analysis of utterance. For each utterance, F0 values <75 hertz and intensity values <20 decibels were excluded from the analysis as they could be a sign of hesitation. This was also manually checked and removed by the researcher. Also, data that are out of 95% CI was removed before each analysis.

### Statistical Analysis

Data on 11 acoustic parameters were evaluated for normality using the Kolmogorov–Smirnov test and showed a nonnormal distribution. Consequently, between-group comparison (for pretraining and posttraining data) was performed using a nonparametric Wilcoxon signed-rank test. All analyses and data visualizations were made on a statistical program RStudio 2024.12.0 Build 467.

### Ethics Approval

This study was reviewed and approved by the institutional review board (IRB) of the Faculty of Medicine, Chulalongkorn University, Bangkok, Thailand. The approval was granted under COA number 1160/2023 and IRB number 577/64. All research activities were conducted in full compliance with international guidelines for human research ethics. Written informed consent was obtained from all participants before their participation. All data collected were anonymized and kept confidential. Participants received compensation of 500 THB (US $15), which covered their participation in this study and other related components. Participation was entirely voluntary, and care was taken to ensure that there was no coercion or undue influence

## Results

### Overview

Analysis of acoustic data from pre- and post-TeamSTEPPS training sessions revealed significant differences in several acoustic parameters. Those parameters are reported in the following subsection. Note that only results from medical and nursing students are accompanied by visualization in the section owing to their significant differences in several acoustic parameters.

### Medical Students

**Figure 2:**
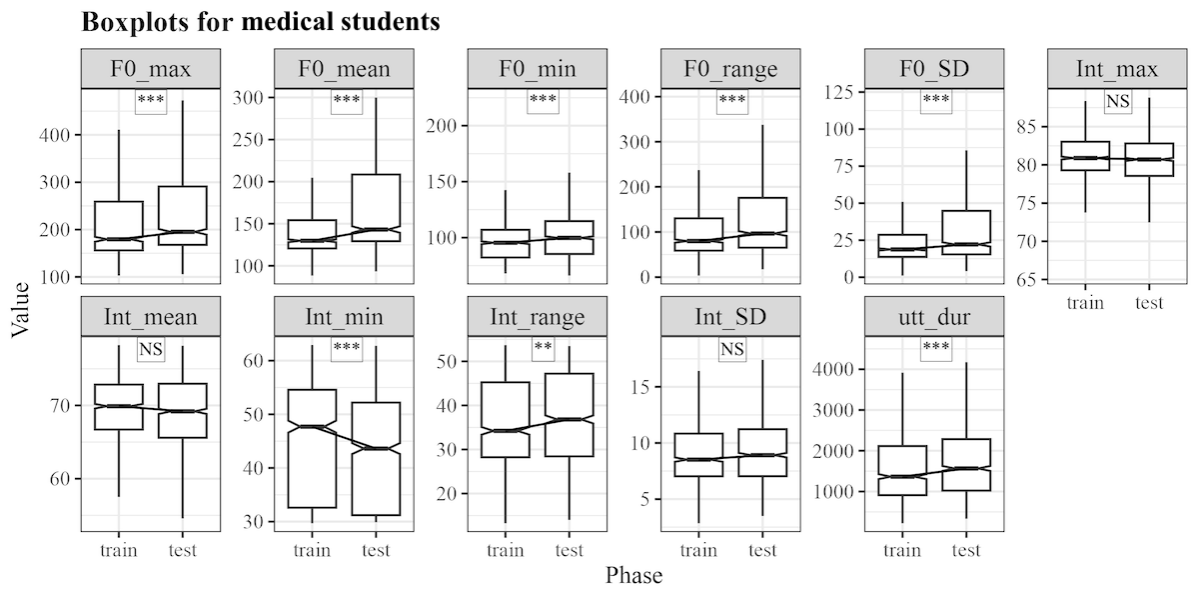
Comparison of acoustic parameters between pretraining (train) and posttraining (test) of medical students. *P<.05; **P<.01; ***P<001. NS: nonsignificant.

Figure 2 shows that pretraining and posttraining data of medical students revealed significant differences between the median in utterance duration, where W represents the test statistic from the Wilcoxon Signed Rank Test, (W=772,124.5; *P*<.001), maximum of F0 (W=831,337; *P*<.001), a minimum of F0 (W=790,596; *P*<.001), mean of F0 (W=936,397; *P*<.001), SD of F0 (W=826,021; *P*<.001), range of F0 (W=807,093; *P*<.001), minimum of intensity (W=646,219; *P*<.001), and range of intensity (W=752,104; *P*=.003). After the TeamSTEPPES training, the medical students spoke with a significantly higher pitch in the posttraining session.

### Nursing Students

**Figure 3:**
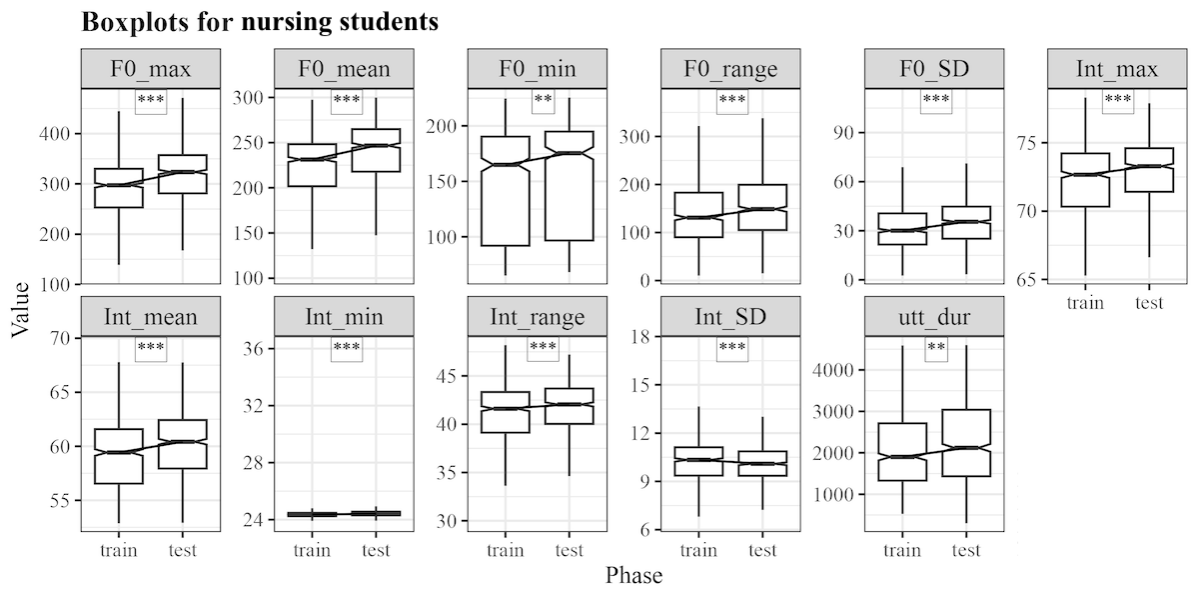
Comparison of acoustic parameters between pretraining (train) and posttraining (test) of nursing students. *P<.05; **P<.01; ***P<001. NS: nonsignificant.

Figure 3 shows that pretraining and posttraining data of nursing students revealed significant differences in the median of utterance duration (W=573,097.5, *P*<.001), maximum of F0 (W=640,250; *P*<.001), a minimum of F0 (W=550,513; *P*=.005), mean of F0 (W= 639,980; *P*<.001), SD of F0 (W=589,489; *P*<.001), range of F0 (W=594,537; *P*<.001), maximum of intensity (W=614,402; *P*<.001), minimum of intensity (W=622,187, *P*<.001), mean of intensity (W=634,827; *P*<.001), and range of intensity (W=601,354; *P*<.001). Nursing students showed a significant increase in the pitch of their speech in the posttraining session; their loudness also significantly increased, as suggested by the data (Figure 3).

Pretraining and posttraining data of medical technology students (Multimedia Appendix 1) showed significant differences in the median of utterance duration (W=36,074; *P*=.004), maximum of F0 (W=27,464; *P*=.01), mean of F0 (W=27,326; *P*=.01), maximum of intensity (W=27,392; *P*=.01), intensity minimum (W=26,400; *P*=.001), maximum of intensity (W=27,110; *P*=.007), and SD of intensity (W=35,381; *P*=.015). Results suggest that medical technology students spoke with significantly quieter and significantly deeper voices after the TeamSTEPPES training.

Pretraining and posttraining data of radiological technology students (Multimedia Appendix 2) showed significant differences in the median of a maximum of F0 (W=5120, P=.002), mean of F0 (W=5169, *P*=.002), SD of F0 (W=4893; *P*=.02), and range of F0 (W=4895; *P*=.02). Data suggests that radiological technology students spoke with significantly deeper voice after the training, as the mean of F0 decreased.

Pretraining and posttraining data of pharmacy students (Multimedia Appendix 3) revealed a significant difference only in the median of minimum of intensity (W=29,799; *P*=.02).

**Figure 4:**
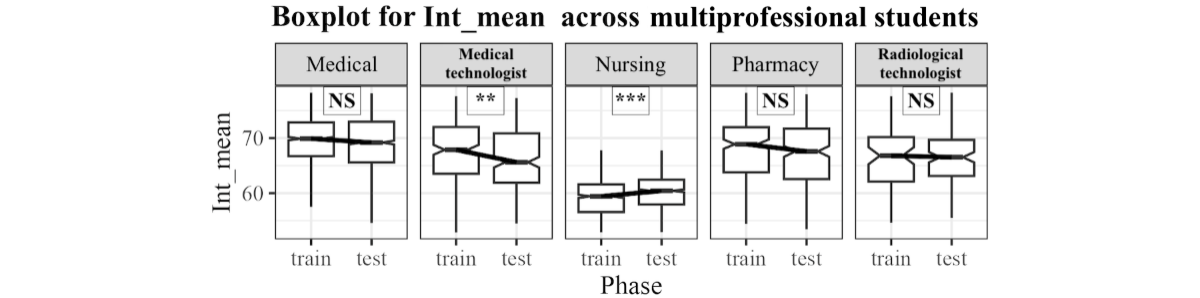
Comparison of the distribution of intensity between pretraining (train) and posttraining (test) of the participant groups. *P<.05; **P<.01; ***P<001. NS: nonsignificant.

Figure 4 shows that data on medians of the averaged intensity showed significant differences between nursing and medical technology students. A significant increase in intensity was noted, indicating louder voices, from the pretraining phase (58.0569754) to the posttraining phase (60.027715) among nursing students (W=634,827; *P*<.001). Conversely, medical technology students showed a significantly decreased intensity, indicating quieter voices, from the pretraining phase (68.1111611) to the posttraining phase (66.033169; W=27,110; *P*=.007).

**Figure 5:**
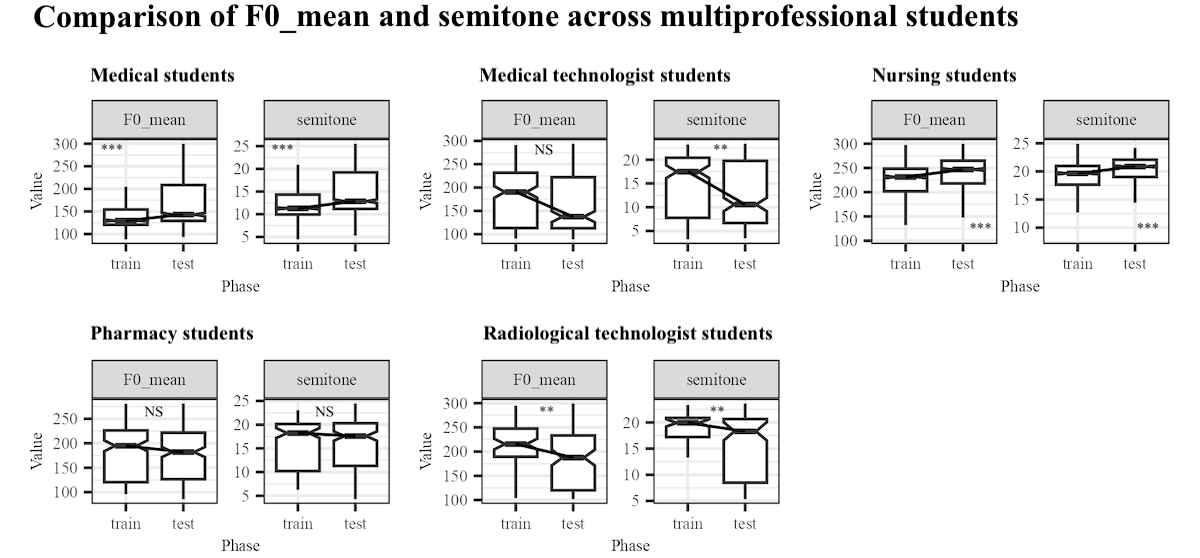
Comparison of the distribution of fundamental frequency and semitone between pretraining (train) and posttraining (test) of the participant groups. *P<.05; **P<.01; ***P<001. NS: nonsignificant.

Figure 5 shows that data on medians of the averaged fundamental frequency showed a significant difference in all student groups except for pharmacy students. Significant increases were observed in the median of the mean of F0 among nursing students, from 230.668 in the pretraining phase to 247.085 in the posttraining phase (W=639,980; *P*<.001), and medical students, from 131.446 in the pretraining phase to 146.014 in the posttraining phase (W=936,397; *P*<.001). This significant increase in pitch among nursing and medical students shows that they spoke with lighter voices. Conversely, radiological technology students showed a significantly decreased fundamental frequency from the pretraining phase (212.175) to the posttraining phase (177.072; W=5066; *P*=.004), and medical technology students showed a similar trend, with mean of F0 in the pretraining phase (198.468) showing a significant decrease in the posttraining phase (139.219; W=27,326; *P*=.01). These significant reductions in pitch show that radiological and medical technology students spoke with deeper voices after the TeamSTEPPES training. Test statistics for all comparisons of acoustic parameters from all student groups are presented in Multimedia Appendix 4.

F0 is also related to different genders. To minimize the effect of biological sex on F0, the F0 was normalized by converting it into semitones. The formula used for this normalization is:



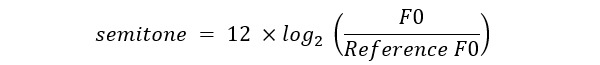



Where the reference value is the individual's minimal F0. As shown in Figure 5, the semitones confirm the results of the mean of F0, supporting the conclusion that the observed differences are not due to gender differences.

### Toward Politeness

Jitwiriyanont and Saisuwan [17] stated that politeness is perceived in speech through pitch variability in individuals across social groups. The results of this study showed a significant increase in pitch variability among medical (W=589,489; *P*<.001; Figure 2) and nursing students (W=826,021; *P*<.001; Figure 3). This finding aligns with the notion that a higher pitch is frequently associated with liveliness and enthusiasm, characteristics that are essential in professional settings such as health care, where doctors and nurses must communicate attentively and empathetically.

Further supporting this, results showed that politeness in medical (W=936397; *P*<.001) and nursing students (W=639980; *P*<.001) was associated with a significant increase in the mean F0. This increase in pitch suggests that liveliness, which conveys enthusiasm, plays a key role in interactions within these professions. In addition, assertiveness, frequently associated with higher intensity, was associated with a significant increase in intensity among nursing students (W=634,827; *P*<.001). Thus, pitch and intensity contribute to the perception of enthusiasm and politeness, with intensity indicating assertiveness in speech.

## Discussion

### Principal Results

This study examining the impact of the TeamSTEPPS training on various health care students, including medical, nursing, medical technology, radiological technology, and pharmacy students, indicated notable improvements in communication across all groups, as reflected by changes in acoustic parameters.

Medical students showed significantly increased F0 variability and mean of F0, suggesting that their speech became more dynamic, reflecting enhanced confidence and engagement. These students demonstrated extended speech duration and frequent think-aloud behaviors, aligning with leadership roles in the TeamSTEPPS. Their increased pitch variation and energetic communication signaled liveliness, whereas their controlled vocal intensity maintained a professional tone. Specifically, medical students showed significantly increased maximum of F0, mean of F0, and range of F0 (W=831,337, 936,397, and 807,093, respectively, *P*<.001), which may suggest a more dynamic communication style. This change could be related to enhanced confidence or increased verbal participation, potentially reflecting leadership presence in the TeamSTEPPS scenario. However, other factors, such as familiarity with the simulation environment, may also have contributed to this change.

Similarly, nursing students exhibited significantly increased pitch and intensity, particularly in the mean of F0 and mean of intensity (W=639,980 and 634,827, respectively, *P*<.001). The livelier tone could be interpreted as a sign of greater engagement and assertiveness. However, it is important to consider that this change might also be due to factors such as growing familiarity with the simulation context or general increases in verbal confidence. Their higher pitch and increased intensity underscored improved confidence and clearer communication, fully aligning with the TeamSTEPPS principles of assertiveness and effective teamwork. Debriefing in a psychologically safe environment has been shown to enhance resilience, reduce stress, and improve the emotional well-being of health care workers [19]. This supportive setting likely contributed to nursing students’ increased confidence and encouraged more active engagement in patient-centered communication, ultimately improving their overall communication skills and confidence in clinical settings.

In contrast, medical technology students displayed decreased pitch and intensity, with significantly decreased mean of F0_and mean of intensity (W=27,326 and 27,110; *P*=.01 and *P*=.007, respectively), suggesting quieter and deeper speech posttraining. This change likely reflects a more composed and controlled communication style.

The utterance duration is a key parameter for understanding the length and structure of communication. A significant increase in duration may indicate a more intricate and detailed exchange of information during the test round. The significant increase in utterances among doctors, nurses, and medical technologists can be attributed to more detailed patient identification information, including frequent identification of names and professional designations, as well as critical details related to medication safety, radiological concerns, and reporting critical values. This approach ensures that all relevant pieces of information are accurately communicated and understood, enhancing overall patient safety and effective teamwork. Furthermore, team members engage in more frequent closed-loop communication and verification, along with increased reminders and sharing of critical patient information to enhance situational awareness. In addition, more mutual support and expressions of gratitude are exhibited.

Similarly, radiological technology students exhibited a significantly decreased pitch, with reductions in the mean of F0 and range of F0 (W=5066 and 5319; *P*=.004 and *P*=.02, respectively), indicating a more deliberate and measured form of speech posttraining.

Pharmacy students showed less pronounced changes, with a notable decrease in minimum of intensity (W=29,799; *P*=.02), suggesting a subtler approach in their posttraining.

Overall, the results suggest that the TeamSTEPPS training enhanced communication among health care students, particularly increasing politeness among medical students and both politeness and assertiveness among nursing students while promoting more controlled and subtler speech among medical technology and radiological technology students. This finding highlights the training’s effectiveness in adapting communication styles to fit the distinct needs of each professional role, fostering interprofessional collaboration and role-specific improvements in communication.

### Limitations

The virtual simulation-based interprofessional design was challenging and limited communication opportunities for medical technologists, radiological technologists, and pharmacists, thereby resulting in fewer observed improvements in their tone of voice [20]. Analyzing communication among pharmacy, medical technology, and radiological technology students presents challenges owing to the significant differences in communication volume compared with physicians and nurses. The number of spoken words (tokens) was markedly lower among pharmacists (9.2%), medical technologists (8.9%), and radiological technologists (4.2%). This decreased token count reflected their supplementary roles and limited involvement in the simulation compared with the primary roles of physicians and nurses. Future research should include equal roles for participants of different professions. In addition, in this study, we did not include a control group because this is a novel research area, and the study was conducted during the COVID-19 pandemic, which posed significant logistical and ethical challenges in study design.

### Comparison With Previous Work

This study adds to existing research by demonstrating that the TeamSTEPPS virtual simulation IPE training effectively enhances team communication, particularly in the areas of politeness and assertive communication across healthcare students, including medical, nursing, pharmacy, medical technology, and radiological technology students. According to [17], in Thai society, overall pitch and pitch variability are crucial for the perception of proper and polite speech. Subsequently, our study demonstrates that the mean of F0 (overall pitch) and SD of F0 (pitch variability) are indicators associated with the perception of proper speech in the context of interprofessional communication.

A previous study by Bertagni et al [21] has highlighted the significance of speech analysis in medical simulation, emphasizing the role of prosodic features including pitch and intensity in shaping the perceptions of confidence and emotional regulation during crisis situations. The present study builds on these findings by showing how the TeamSTEPPS training can fine-tune these acoustic features, particularly pitch variability and intensity, thereby leading to more effective and nuanced communication. Moreover, the inclusion of diverse professional groups in this study broadens the scope of previous studies, providing a more comprehensive understanding of how IPE impacts communication dynamics across various healthcare roles. In addition, although Bertagni et al [21] focused on real-time speech analysis using tools, including e-REAL, for tracking verbal and nonverbal aspects of communication, this study goes further by applying acoustic analysis for assessing long-term changes in communication patterns posttraining. This integration of speech analysis into IPE contributes valuable insights into how training can improve not only verbal assertiveness but also emotional intelligence and team cohesion, which are critical in emergency health care environments.

Health care education institutions must be committed to developing professional standards, making it essential for health care professionals to undergo IPE programs [22]. These programs allow students to practice and hone their skills before working with actual patients. Research has shown that Sim IPE and TeamSTEPPS [23] are effective tools for enhancing teamwork and communication skills among health care students [24,25]. In addition, a scoping review has reported that virtual simulation can be as effective as face-to-face simulation and standardized patients in teaching team communication, providing optimum stress levels, and offering scalability [26,27].

Currently, global research on the tone of voice, particularly in professions outside of medicine and nursing, such as pharmacy, radiological technology, and medical technology, is limited [28]. This is especially true in emergency situations, wherein courage is required to speak up for patient safety [29]. Although some studies, including those by Morrow et al [30], have addressed this issue, this study does not provide a detailed acoustic analysis. Further investigation into acoustic analyses would enhance our understanding of the role of prosodic cues in interprofessional communication.

From another perspective, research on politeness in different languages has revealed varied patterns. Generally, studies have reported that polite speech frequently involves higher pitch [31,32]. However, in some languages, such as Korean, polite speech is associated with lower pitch [33]. In Japan, findings are less consistent; although some studies have indicated that polite speech involves higher pitch [34], others have shown no significant relationship between pitch level and politeness [14].

This study extends the findings of Jitwiriyanont and Saisuwan [17] by confirming that pitch variability, rather than overall pitch height, plays a crucial role in conveying politeness in Thai. Specifically, the study found that increased SD of F0 and a wider range of F0 are associated with perceived politeness, particularly among medical and nursing students. This indicates that dynamic pitch modulation, rather than a consistently high pitch, contributes to a polite and attentive communication style. In addition, the study observed that younger Thai speakers, especially in professional settings, tend to use a more varied pitch pattern, reflecting liveliness and respect. These acoustic characteristics align with cultural norms in Thai society, where dynamic vocal delivery is perceived as more attentive and respectful, enhancing the effectiveness of interprofessional communication [17].

### Future Study Recommendation

To improve team-wide communication and prepare students for real-world interprofessional collaboration, future virtual simulation IPE should prioritize equal participation and role balance across healthcare professions. Research should further explore virtual simulations as scalable tools for IPE, investigating their long-term effects on communication dynamics. In practice, incorporating these TeamSTEPPS virtual simulation IPE programs into standard health care curricula will enhance team dynamics, leadership, and patient care, especially in crises. At the policy level, a broader adoption of standardized virtual simulation IPE programs focused on communication competencies could foster more cohesive teams and improve patient outcomes across health care settings. This initiative could represent a significant advancement in educational innovation, providing scalable methods for overcoming challenges in measuring and providing effective feedback to improve the tone of voice among interprofessional health care students. Finally, we acknowledge the importance of a more rigorous comparative approach. Therefore, we propose that future research should employ a randomized controlled trial.

### Conclusions

The critical role of the tone of voice and prosodic features in enhancing communication skills across health care professions through the TeamSTEPPS virtual simulation IPE training is highlighted in this study. Medical and nursing students demonstrated significant improvements in politeness and assertiveness, whereas medical technology, radiological technology, and pharmacy students experienced more subtle gains, frequently reflected in their quieter more controlled communication. These improvements extend beyond word choice to key vocal elements, including pitch, loudness, and duration, ensuring that communication is perceived as respectful, empathetic, and clear. To prevent misunderstandings and foster effective and compassionate communication in health care settings, mastery of prosody is significant.

The findings underscore the significance of incorporating virtual simulation IPE into health care curricula for enhancing teamwork, leadership, and patient-centered care, particularly in crisis situations where effective communication is vital. This study advocates for the broader adoption of standardized IPE programs that emphasize communication competencies, potentially leading to improved interdisciplinary teamwork and better patient outcomes.

## Appendix

Multimedia Appendix 1 Comparison of acoustic parameters between pre-training (train) and post-training (test) of medical technology students.

Multimedia Appendix 2 Comparison of acoustic parameters between pre-training (train) and post-training (test) of radiological technology students.

Multimedia Appendix 3 Comparison of acoustic parameters between pre-training (train) and post-training (test) of pharmacy students.

Multimedia Appendix 4 Table of test statistics and p-values for comparisons of acoustic parameters from multiprofessional students.

## Abbreviations

COPD: chronic obstructive pulmonary disease
ED: emergency department
F0: fundamental frequency
IPE: interprofessional education
IRB: institutional review board
SimBIE: simulation-based interprofessional education
TeamSTEPPS: Team and Strategies to Enhance Performance and Patient Safety
VR: virtual reality

## Data Availability

The dataset is publicly available on GitHub [35].

## References

[1] Narajeenron K, Tarapan T, Dukovic J, Sutham K, Imsuwan I, Dadeh A, Nakornchai T, Bengiamin D, Chakravarthy B, Anderson C, Eiam-Ong S, Hoonpongsimanont W (2024). Perceptions of emergency physician professionalism among healthcare providers and patients: A multicenter study in Thailand and the US. Chulalongkorn Medical Journal.

[2] Robinson FP, Gorman G, Slimmer LW, Yudkowsky R (2010). Perceptions of effective and ineffective nurse-physician communication in hospitals. Nurs Forum.

[3] Weller J, Boyd M, Cumin D (2014). Teams, tribes and patient safety: overcoming barriers to effective teamwork in healthcare. Postgrad Med J.

[4] Stecker M (2015). Inter- and intraprofessional respect: A dying concept?. Surg Neurol Int.

[5] Brown P, Levinson SC (1987). 5.1 Construction of ECM-based xenogeneic spleen bionic scaffold.

[6] Mehrabian A (1971). Silent Messages.

[7] Ambady N, Koo J, Lee F, Rosenthal R (1996). More than words: linguistic and nonlinguistic politeness in two cultures. Journal of Personality and Social Psychology.

[8] Trees AR, Manusov V (1998). Managing face concerns in criticism integrating nonverbal behaviors as a dimension of politeness in female friendship dyads. Human Comm Res.

[9] Laplante D, Ambady N (2003). How things are said: voice tone, voice intensity, verbal content, and perceptions of politeness. J Lang Soc Psychol.

[10] Navarro AH, Nebot AC (2014). On the importance of the prosodic component in the expression of linguistic im/politeness. J Politeness Res.

[11] Culpeper J, Bousfield D, Wichmann A (2003). Impoliteness revisited: with special reference to dynamic and prosodic aspects. Journal of Pragmatics.

[12] Wichmann A (2004). The intonation of Please-requests: a corpus-based study. Journal of Pragmatics.

[13] Grawunder S, Winter B (2010). Acoustic correlates of politeness: prosodic and voice quality measures in polite and informal speech of Korean and German speakers. Speech Prosody.

[14] Idemaru K, Winter B, Brown L (2019). Cross-cultural multimodal politeness: the phonetics of Japanese deferential speech in comparison to Korean. Intercul Pragmat.

[15] Leongómez JD, Mileva VR, Little AC, Roberts SC (2017). Perceived differences in social status between speaker and listener affect the speaker's vocal characteristics. PLoS One.

[16] Sorokowski P, Puts D, Johnson J, Żółkiewicz O, Oleszkiewicz A, Sorokowska A, Kowal M, Borkowska B, Pisanski K (2019). Voice of authority: professionals lower their vocal frequencies when giving expert advice. J Nonverbal Behav.

[17] Jitwiriyanont S, Saisuwanditors P (2023). Polite tones of voice in transition: investigating speech production and perception in Thai speakers of different generations.

[18] Boersma P, Weenink D (2001). PRAAT, a system for doing phonetics by computer. Glot Int.

[19] Evans TR, Burns C, Essex R, Finnerty G, Hatton E, Clements AJ, Breau G, Quinn F, Elliott H, Smith LD, Matthews B, Jennings K, Crossman J, Williams G, Miller D, Harold B, Gurnett P, Jagodzinski L, Smith J, Milligan W, Markowski M, Collins P, Yoshimatsu Y, Margalef Turull J, Colpus M, Dayson ML, Weldon S (2023). A systematic scoping review on the evidence behind debriefing practices for the wellbeing/emotional outcomes of healthcare workers. Front Psychiatry.

[20] Benishek LE, Lazzara E, Sonesh S, Paige J, Sonesh S, Garbee D, Bonanno L (2020). Challenges to conducting simulation-based interprofessional education for non-technical skills. Comprehensive Healthcare Simulation: Interprofessional Team Training and Simulation.

[21] Bertagni B, Salvetti F, Gardner R (2022). Speech analysis for advanced medical simulation. Innovations in Learning and Technology for the Workplace and Higher Education: Proceedings of the Learning Ideas Conference?.

[22] Collaborative IE (2023). IPEC core competencies for interprofessional collaborative practice: version 3. IPEC.

[23] Kheawwan P, Thanomlikhit C, Narajeeenron K, Rojnawee S (2024). Translation and psychometric validation of the Thai version of TeamSTEPPS® team performance observation tool. J Interprof Care.

[24] Mahmood LS, Mohammed CA, Gilbert JHV (2021). Interprofessional simulation education to enhance teamwork and communication skills among medical and nursing undergraduates using the TeamSTEPPS® framework. Med J Armed Forces India.

[25] Sezgin MG, Bektas H (2023). Effectiveness of interprofessional simulation-based education programs to improve teamwork and communication for students in the healthcare profession: A systematic review and meta-analysis of randomized controlled trials. Nurse Educ Today.

[26] Liaw SY, Ooi SL, Mildon R, Ang ENK, Lau TC, Chua WL (2022). Translation of an evidence-based virtual reality simulation-based interprofessional education into health education curriculums: an implementation science method. Nurse Educ Today.

[27] Wu Q, Wang Y, Lu L, Chen Y, Long H, Wang J (2022). Virtual simulation in undergraduate medical education: A scoping review of recent practice. Front Med (Lausanne).

[28] Friary P, Purdy SC, McAllister L, Barrow M (2021). Voice behavior in healthcare: A scoping review of the study of voice behavior in healthcare workers. J Allied Health.

[29] Kim J, Gonzalez-Pumariega G, Park S (2023). Urgency builds trust: a voice agent's emotional expression in an emergency.

[30] Morrow KJ, Gustavson AM, Jones J (2016). Speaking up behaviours (safety voices) of healthcare workers: A metasynthesis of qualitative research studies. Int J Nurs Stud.

[31] Chen A, Gussenhoven C, Rietveld T (2004). Language-specificity in the perception of paralinguistic intonational meaning. Lang Speech.

[32] Devís HE, Cantero SF (2014). The intonation of mitigating politeness in catalan. J Politeness Res.

[33] Winter B, Grawunder S (2012). The phonetic profile of Korean formal and informal speech registers. Journal of Phonetics.

[34] Loveday L (1981). Pitch, politeness and sexual role: an exploratory investigation into the pitch correlates of english and Japanese politeness formulae. Lang Speech.

[35] rattanasuwan-r / simipechula. GitHub.

