# Supplementary figures and images for: Advancing Politeness and Assertive Communication Through Tone of Voice in Crisis Team Situations: Pre-Post Acoustic Analysis Study of Team and Strategies to Enhance Performance and Patient Safety (TeamSTEPPS) Virtual Simulation for Interprofessional Education in Health Care Undergraduate Students
Source: J Med Internet Res. 2025 Apr 21;27:e66988. doi: 10.2196/66988 (PMC12052860; doi:10.2196/66988)

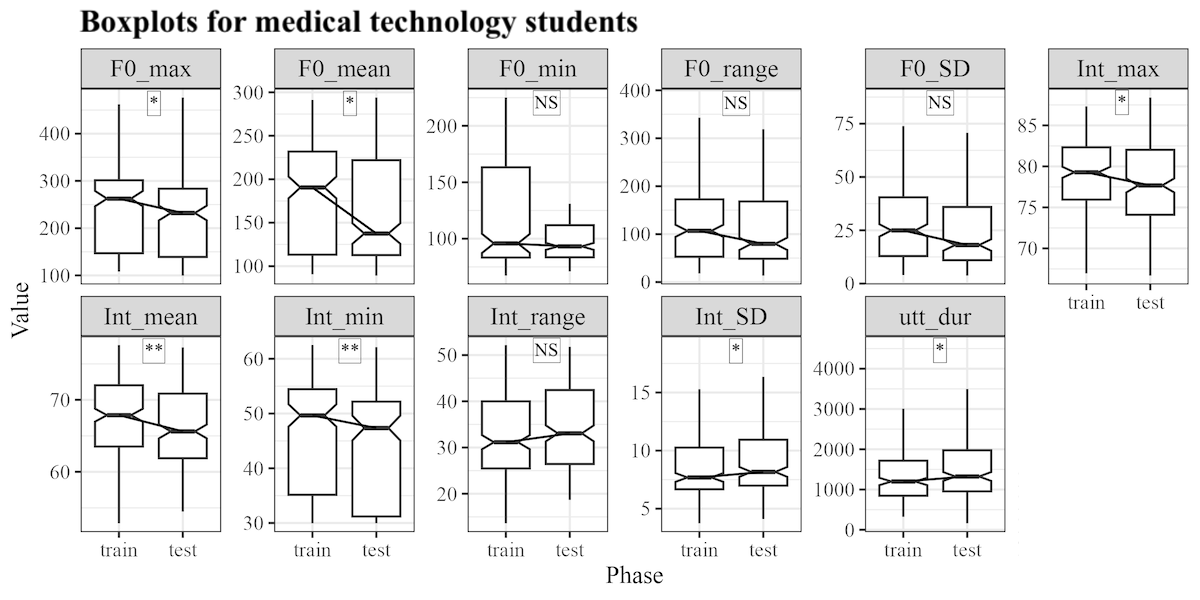

Supplement: Multimedia Appendix 1 [file jmir_v27i1e66988_app1.png]

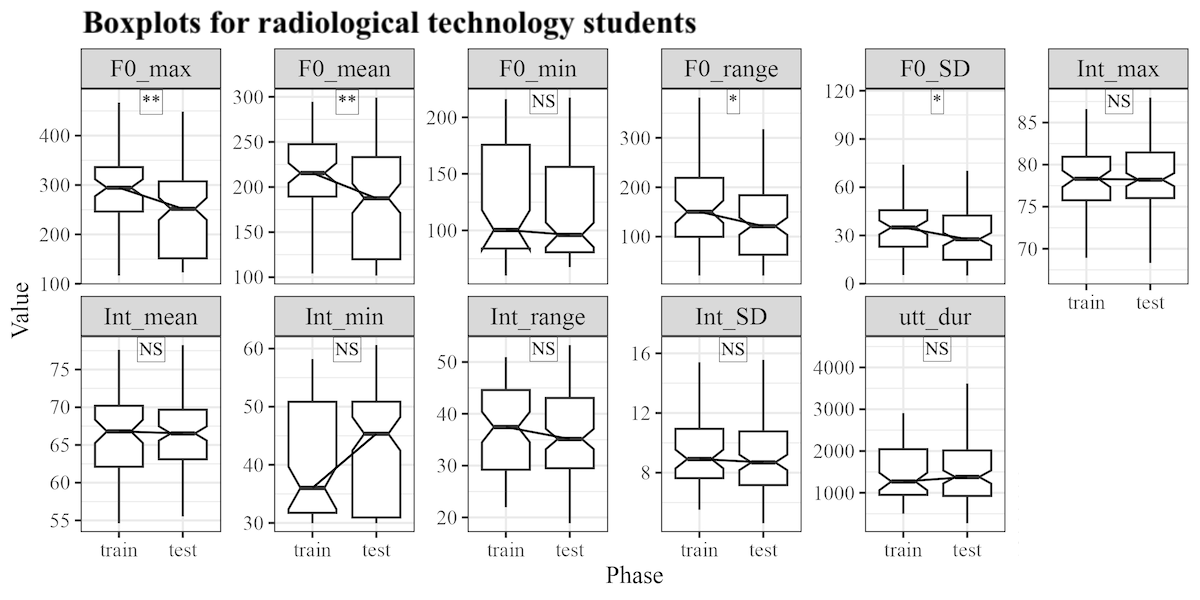

Supplement: Multimedia Appendix 2 [file jmir_v27i1e66988_app2.png]

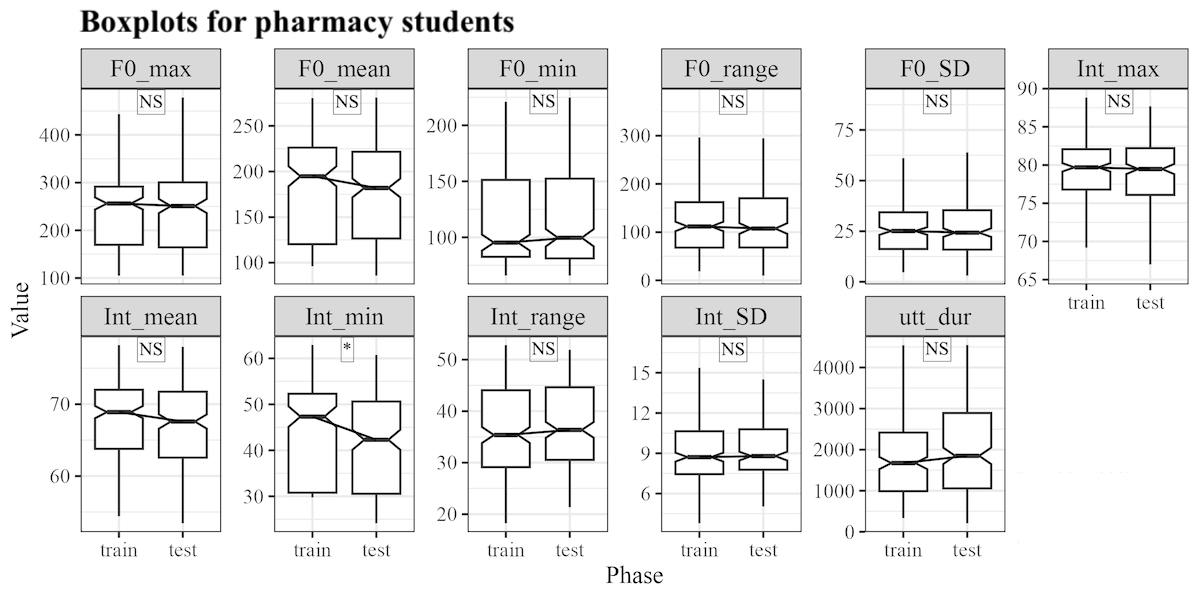

Supplement: Multimedia Appendix 3 [file jmir_v27i1e66988_app3.png]

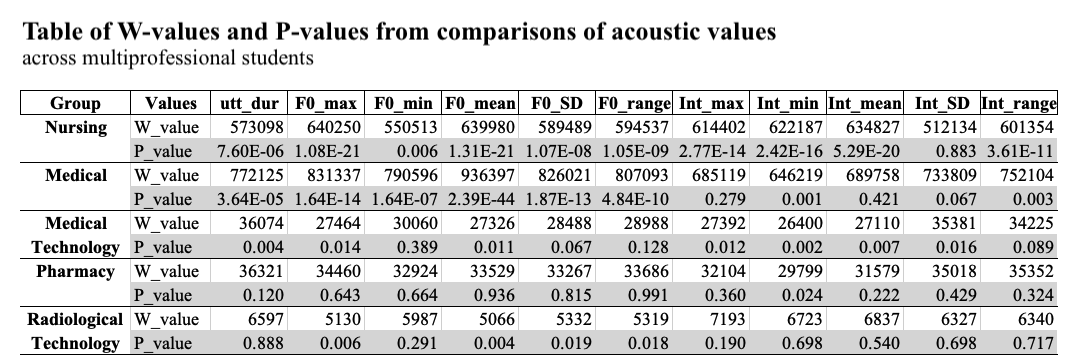

Supplement: Multimedia Appendix 4 [file jmir_v27i1e66988_app4.png]
